# Patients perceived challenges in seeking dental care in Komfo Anokye Teaching Hospital

**DOI:** 10.1371/journal.pone.0325136

**Published:** 2025-06-02

**Authors:** Kwame Adu Okyere Boadu, Richard Okyere Boadu, Esther Priscilla Biamah Danquah, Nana Atuahene Oti, John Billy Owusu Quarshie, Nana Bempong Owusu-Ankomah, Moses Yeboah Addo

**Affiliations:** 1 School of Dentistry, College of Health Sciences, Kwame Nkrumah University of Science and Technology, Kumasi, Ghana; 2 Department of Health Information Management, School of Allied Health Sciences, College of Health and Allied Health Sciences, University of Cape Coast, Cape Coast, Ghana; 3 Faculty of Pharmacy and Pharmaceutical Sciences, College of Health Sciences, Kwame Nkrumah University of Science and Technology, Kumasi, Ghana; Indiana University School of Dentistry, UNITED STATES OF AMERICA

## Abstract

**Background:**

Access to dental care services remains a global challenge, with millions encountering barriers that hinder their utilization, despite the recognized importance of oral health to overall well-being. The intricate relationship between oral health and general well-being highlights the severe impact of oral disorders on global economies and public health systems when access barriers exist. This study aimed to assess the perceived challenges in seeking dental care among patients at the Oral Health Directorate of Komfo Anokye Teaching Hospital.

**Methods:**

This study employed a mixed methods approach, combining both quantitative and qualitative research methods for 130 respondents. With the quantitative approach: it was a cross-sectional design where structured questionnaires were administered to participants and data was analyzed using SPSS IBM version 23. The qualitative approach on the other hand, utilized semi-structured interviews to explore participants’ experiences, perspectives, and challenges related to accessing dental care. Data was analyzed using thematic analysis to identify key themes and patterns within the responses. The study had a response rate of 94.89%.

**Results:**

Most respondents were females (56.2%) and aged 25–34 (26.9%). Approximately 55.4% had encountered barriers, with cost and lack of appointment times being major concerns. Regarding challenges at the dental clinics, lengthy waits (63.2%) and dental anxiety (77.0%) were the most common concerns. Gender and marital status were the only demographic characteristics which were statistically significantly associated with quality of care sought. Gender had a p-value of 0.00, indicating strong evidence of an association, while marital status had a p-value of 0.055, suggesting moderate evidence of an association. Marital status and education were found to be statistically significantly associated with perceived treatment-seeking behaviours, with p-values of 0.012 and 0.009 respectively. Postponement of dental care results in deteriorating oral health, higher expenses, and emotional strain for patients. The study identified long wait times, unclear communication, and financial barriers as key challenges. Patients proposed solutions including streamlined appointments, jargon-free explanations, and more affordable care options to improve dental experiences and accessibility.

**Conclusions:**

Over half of respondents faced obstacles like cost and limited appointment availability. Patients also encountered challenges during dental visits, such as anxiety and long wait times. Demographics like gender, marital status, and education were linked to treatment-seeking behavior, suggesting potential disparities in access and utilization. Overall, the study highlights the complex interplay between socio-demographic factors, perceived barriers, patient experiences, and suggestions for enhancing dental care accessibility and quality.

## Introduction

Oral health is a vital component of overall well-being, yet millions worldwide face significant barriers to accessing dental care services [1]. The importance of dental care cannot be overstated as maintaining healthy teeth and gums is crucial not only for proper nutrition but also for one’s self-esteem, social interactions, and overall quality of life [2]. General health and well-being are closely linked to oral health. Oral disorders significantly impact global economies and public health while severely reducing the quality of life for affected individuals [3]. Despite global improvements in oral health over recent decades, dental illnesses, particularly dental caries and periodontal disease remain highly prevalent in underdeveloped nations. According to the Global Burden of Disease survey, oral diseases impacted 3.9 billion persons [4]. The two most common oral disorders worldwide are dental caries (tooth decay) and periodontal disease. The prevalence of periodontal disorders was at least 50% worldwide, and 11.2% of people had severe periodontitis [5]. Similarly, dental caries, affects around 2.4 billion people while early childhood caries, a silent global epidemic affects 621 million children, having a detrimental effect on their quality of life and well-being [4].

Dental issues, when left untreated, can lead to severe pain, infections, and even systemic health problems [2]. Despite the critical role dental care plays in our lives, many individuals encounter obstacles that prevent them from accessing timely and appropriate oral healthcare services. These barriers, whether real or perceived, can have profound consequences for individuals, communities, and public health systems [6]. According to literature, people in impoverished nations frequently report that, dental care services are not used to their full potential [3]. Comprehensive national dental care systems have increased coverage and decreased or even eliminated socioeconomic disparities in the use of dental services in many nations [7] However, disparities in socioeconomic level result in uneven dental service consumption in many nations. The accessibility of dental services, dental anxiety, and expense are among additional barriers to accessing regular oral health care [8].

Consequently, the barriers preventing individuals from accessing oral healthcare services have broad-ranging implications for public health and the overall healthcare system [9]. Efforts to address these perceived barriers to seeking oral healthcare are critical to improving oral health equity and overall public health. Although the various challenges or barriers influencing access to oral care is documented extensively in literature, there is minimal research on such perceived challenges among the Ghanaian population. This therefore exists as a gap in literature. This study therefore seeks to assess the perceived challenges in seeking dental care among patients in the Komfo Anokye Teaching Hospital (KATH).

## Methods

### Study design

This cross-sectional study collected data from patients at the KATH Oral Health Directorate Outpatient Department between October 30th and November 10th, 2023. The study employed a mixed-methods approach, combining both qualitative and quantitative data collection methods.

### Profile of study area

This research was carried out in Komfo Anokye Teaching Hospital (KATH), Kumasi. It is the main referral for inhabitants in the Ashanti, Bono and Ahafo regions mainly. It has a 1200 bed capacity. The Oral Health Directorate is made up of oral diagnostics, restorative dentistry, pediatric and orthodontic dentistry and oral and maxillofacial surgery.

### Study population

The study population involved all patients who were expected to visit the dental clinic at Komfo Anokye Teaching Hospital (KATH) based on the projected population of 20,000 (KATH annual report, 2020). All patients seen in the dental facility were included in both the qualitative and quantitative data collection phases.

### Sample size determination

A total sample size of 137 was calculated using EpiInfo 7 software’s StatCalc function, with parameters: 90% confidence level, 50% expected frequency, 5% acceptable margin of error, design effect of 1, and cluster of 1, based on an estimated population of 20,000. However, only 130 of the 137 partook in this study, yielding a response rate of 94.9%.

### Sampling procedure

A systematic sampling technique was used to select every fifth outpatient (in the order in which they arrived during the day), till respondents reached 14. Questionnaires were handed to participants who met the inclusion criteria at the record section, where they retrieve their cards and are directed to the various units. This process continued until the required respondents were sampled. Only patients with a history of at least one prior visit to the dental facility before the study began were included. Patients who had visited the dental facility for the first time (during this study) or were in a critical condition or unconscious were excluded from the study.

### Data collection and analysis: Quantitative method

Structured questionnaires were employed for the quantitative part. This instrument consisted of a predetermined set of closed-ended questions designed to gather demographic information, assess perceived barriers to accessing dental care, and measure the impact of these barriers on individuals’ oral health behaviors. Approximately 14 participants were recruited and interviewed daily over a 10-day period.

### Validity and reliability: Quantitative method

To ensure the validity and reliability of the questionnaire, the following steps were taken:

i **Content Validity:** The questionnaire was reviewed by experts in the field of oral health to ensure that the questions accurately measured the intended constructs.
ii **Face Validity:** The questionnaire was pilot-tested in Suntreso Hospital for a 3 day period with 20 individuals similar to the target population to assess its clarity and relevance.
iii **Internal Consistency:** The questionnaire was analyzed for internal consistency using Cronbach’s alpha, a measure of the reliability of multiple-item scales. The alpha coefficient for the questionnaire was found to be [0.8], indicating a high level of internal consistency.

### Data collection and analysis: Qualitative method

Semi-structured interviews served as the primary data collection tool for the qualitative portion of the study. This method employed a predetermined set of open-ended questions, providing flexibility to explore additional topics as the interviews progressed. The interviews were conducted in person, audio recorded and transcribed verbatim. Approximately 14 participants were recruited and interviewed daily over a 10-day period.

### Validity and reliability: Qualitative method

i **Interviewer Training:** Interviewers were trained to establish rapport with participants, probe for deeper insights, and avoid leading questions.
ii **Member Checking:** Participants were given the opportunity to review and provide feedback on their transcripts to ensure accuracy and completeness.
iii **Intercoder Reliability:** Multiple researchers analyzed a subset of interviews to assess intercoder agreement on identified themes and categories. A high level of intercoder reliability was achieved, indicating the consistency of the analysis process.
iv **Triangulation:** Qualitative data was triangulated with quantitative findings to provide a more comprehensive understanding of the research topic.

### Qualitative phase

To explore the participants’ experiences and perspectives in depth, semi-structured interviews were conducted. These interviews allowed for open-ended questions and follow-up inquiries, providing a rich source of qualitative data. Participants were recruited from the study population and interviewed individually in a private setting. The interviews were audio-recorded and transcribed verbatim. A thematic analysis approach was employed to identify recurring themes and patterns within the data. These themes were then categorized and analyzed to gain a deeper understanding of the participants’ experiences. To provide a comprehensive overview of the findings, composite studies were created. These studies combined excerpts from multiple participants who shared similar experiences and perspectives [13]. This approach allowed for the identification of important factors and unique characteristics that may not have been captured in individual verbatim quotes.

### Ethical considerations

Ethical clearance (CHRPE/AP/965/23) was granted from the Committee on Human Research, Publications, and Ethics of the KNUST School of Medicine and Dentistry. Informed consent was also obtained from participants. Names of respondents were not collected which warranted anonymity and discretion. Verbal consent was gained from participants in languages they were comfortable with. Confidentiality and the right to pull out from this study at any time were reiterated to respondents before administering questionnaires.

## Results

### Sociodemographic background of respondents

Approximately 56.2% of respondents were females. The most represented age group was 25–34 years (26.9%), while the least represented was 65 years and older (6.9%). More than half (55.4%) of respondents were married and 22.3% of respondents were either in Senior High School or vocational institutions. One-third of respondents were in other occupations rather than being unemployed, salaried workers, housewives, farmers, traders or artisans. Four-fifths of respondents were Christians with the remaining one-fifths being Muslims, Traditionalists or belonging to a different religion was not mentioned. In all, there were 130 respondents for this study as shown in Table 1.

**Table 1:** Socio-demographic characteristics of respondents (n = 130).

Characteristic	Number (%)
Sex	
Male	57(43.8)
Female	73(56.2)
Age	
<15	21(16.2)
15-24	25(19.2)
25-34	35(26.9)
35-44	18(13.9)
45-54	12(9.2)
55-64	10(7.7)
65+	9(6.9)
Marital Status	
Married	47(36.1)
Single	72(55.4)
Divorced/Separated	4(3.1)
Widow/widower	7(5.4)
Education Level	
Post-Secondary/Tertiary	23(17.7)
Senior High School/Vocational	29(22.3)
Junior High School/Middle School	24(18.5)
Primary	26(20.0)
No education	25(19.2)
Others	3(2.3)
Occupation	
Unemployed	21(16.2)
Salaried worker	11(8.5)
Housewife	7(5.4)
Farmer	15(11.4)
Trader	23(17.7)
Artisan	10(7.7)
Other	43(33.1)
Religion	
Traditional	4(3.1)
Christian	106(81.5)
Muslim	18(13.9)
Others	2(1.5)

*Source: Survey, 2023.*

### Perceived challenges that dental patients face before seeking dental care

A **Perceptions of Barriers to Dental Care**

**Fig 1:**
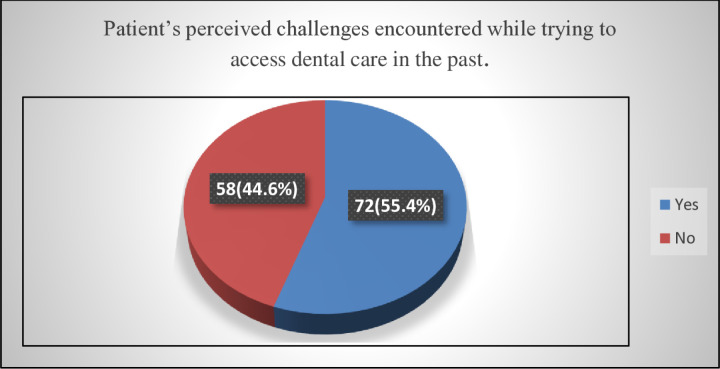
Patient’s perceived challenges encountered while trying to access dental care in the past.

While 55.4% of respondents admitted to encountering challenges/barriers, 44.6% of respondents had never encountered any challenges/barriers while trying to access dental care in the past [Fig 1].

**Fig 2:**
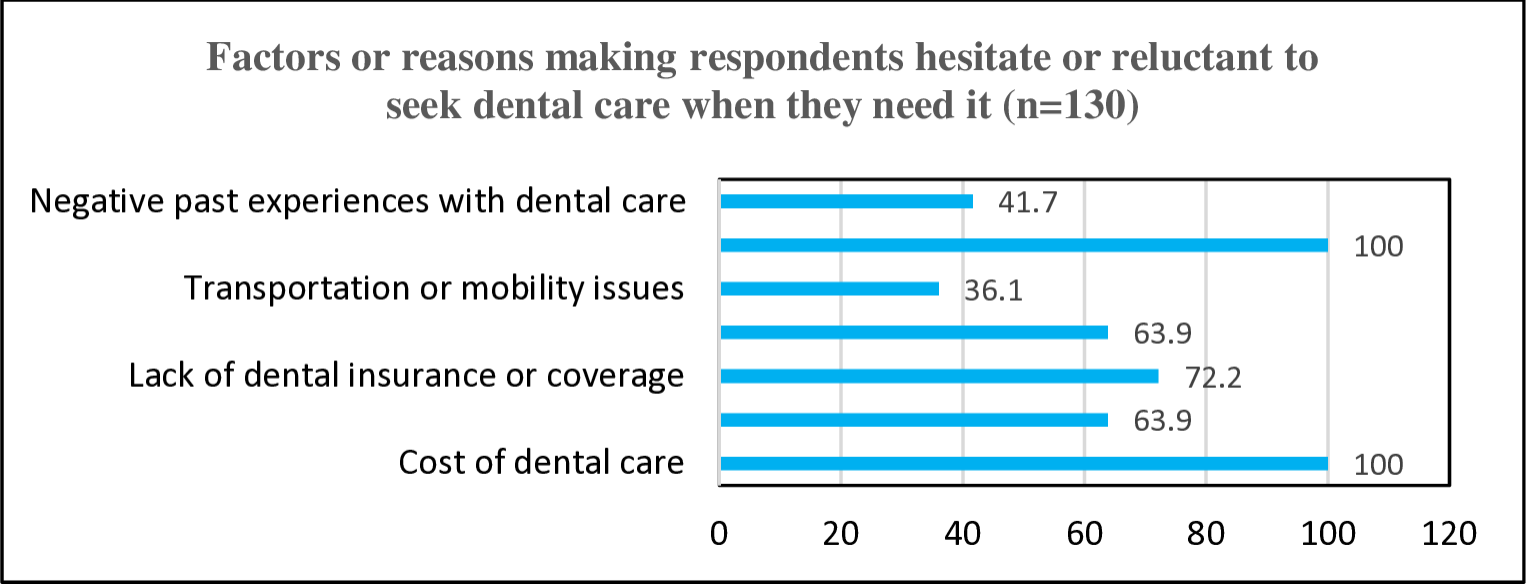
Patient’s perceived factors that make them hesitate to seek dental care.

Participants cited cost (100%), appointment availability (100%), insurance coverage (72.2%), anxiety (63.9%), accessibility (63.9%), past experiences (41.7%), and mobility (36.1%) as significant barriers to accessing dental care [Fig 2].

**Fig 3:**
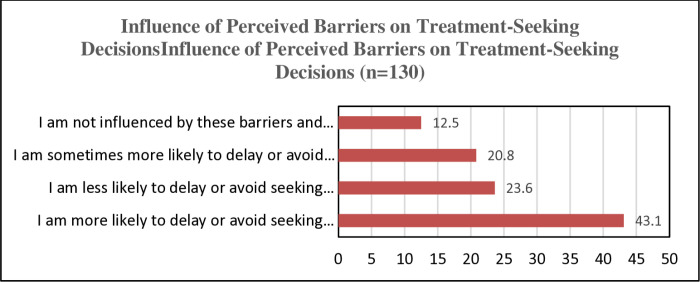
Influence of Perceived Barriers on Treatment-Seeking Decisions.

Despite facing multiple barriers, 43.1% of respondents were more likely to avoid or delay dental care, while 23.6% were less likely to. Approximately 20.8% were sometimes more likely to delay care, and 12.5% were unaffected [Fig 3].

### Perceived challenges that dental patients may face during their visits to the dental clinic

A **Dental Care Seeking Behavior**

**Fig 4:**
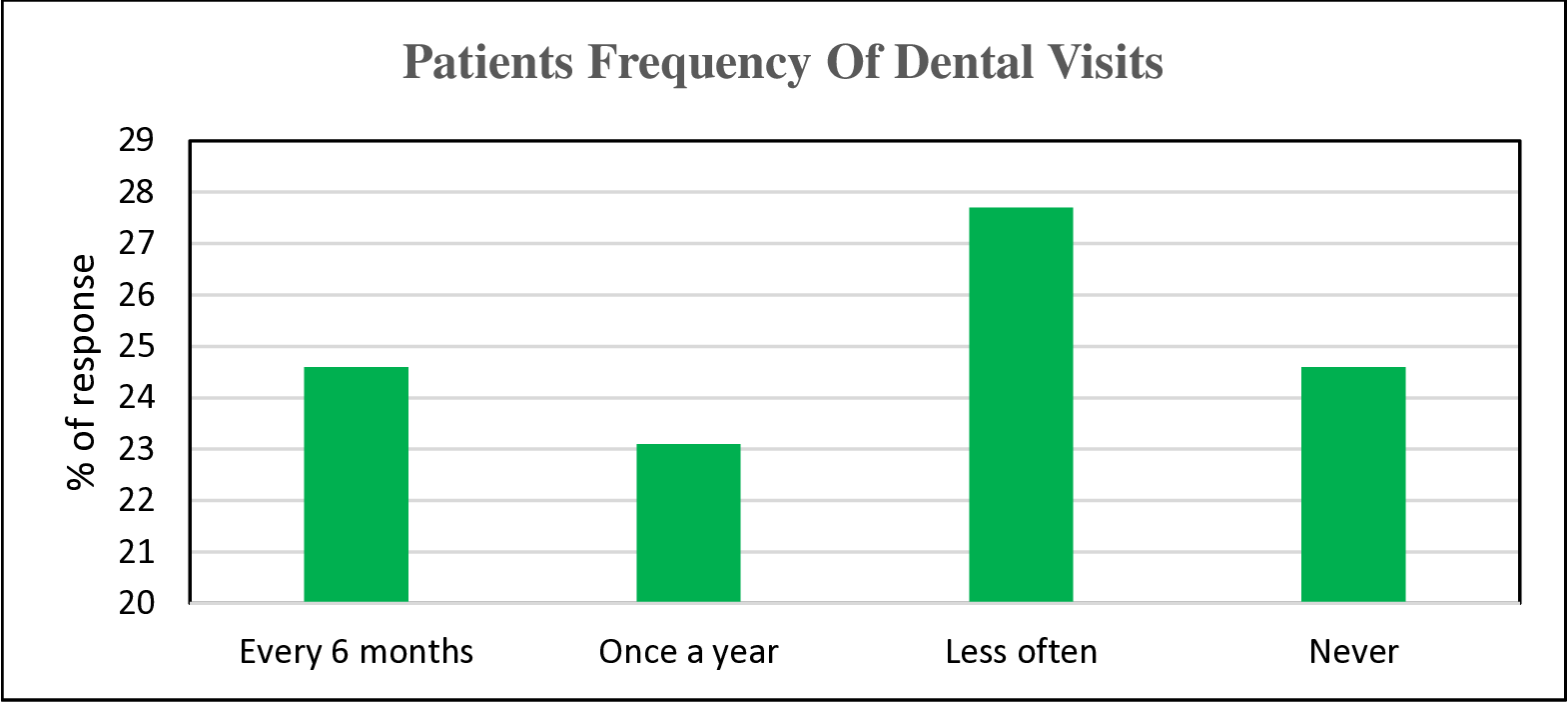
Patient’s frequency of dental visits.

On how often respondents visited the dentists for either check-up or cleaning, 32(24.6%) each of respondents either visited the dentist every 6 months or not at all. While 30(23.1%) visited the dentist once every year, 36(27.7%) visited the dentist less often [Fig 4].

B **Perceived Challenges at the Dental Clinic**

**Fig 5:**
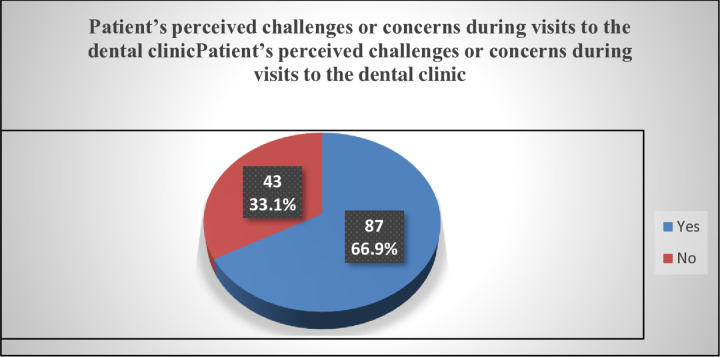
Patient’s perceived challenges or concerns during visits to the dental clinic.

About 87(66.9%) of respondents had encountered a challenge during their visit to the dental clinic while 43(33.1%) had never encountered any challenge during such visits [Fig 5].

Participants reported dental anxiety (77%), lengthy wait times and privacy issues (63.2%), limited appointment availability and high costs (60.9%), discomfort during procedures (41.4%), inadequate communication (40.2%), and lack of transparency (20.7%) as significant challenges faced during their dental visits [Table 2].

**Table 2:** Main challenges or concerns respondents have encountered at the dental clinic (n = 130).

**Variable**	**Frequency**	**Percent**
Lengthy waiting times	55	63.2
Lack of appointment availability	53	60.9
Dental anxiety or fear	67	77.0
Inadequate communication with dental staff	35	40.2
Discomfort during dental procedures	36	41.4
Lack of privacy during treatment	55	63.2
High cost of dental treatment	49	56.3
Lack of transparency in treatment options and costs	18	20.7
Negative interactions with dental staff	55	63.2
Hygiene and cleanliness concerns	49	56.3

*Source: Survey, 2023.*

C **Communication and Transparency**

**Fig 6:**
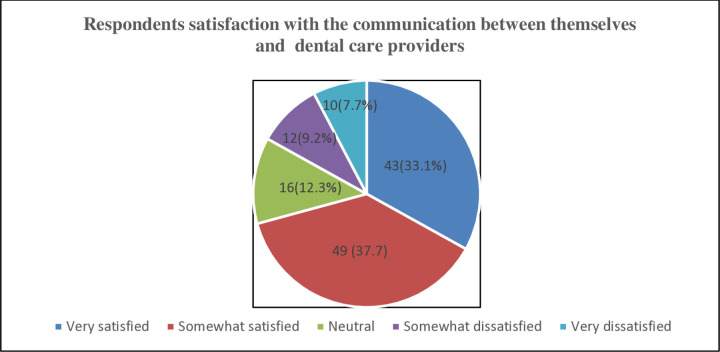
Respondents satisfaction with the communication between themselves and dental care providers. NOTE: The next part of the results captures all the qualitative analysis as seen in the qualitative phase below.

On communication between respondents and dental healthcare providers, 43(33.1%) of respondents were very satisfied, 49(37.7%) were somewhat satisfied, 16(12.3%) were neutral, 12(9.2%) were somewhat dissatisfied and 10(7.7%) were very dissatisfied [Fig 6].

A **Effects Of Delayed Dental Care on Patients Due To Perceived Barriers**

Based on responses from patients, four themes were generated for this qualitative analysis. This part of the composite study looked at how perceived barriers affected oral health and overall well-being.

i **Worsening Oral Health Conditions**: Delaying or avoiding dental care leads to the progression of oral health issues. What could have been addressed as a small concern, like a cavity, worsens and can lead to infections or severe dental complications, potentially resulting in the need for more extensive and costly treatments.
ii **Financial Implications:** Procrastination in seeking dental care often leads to an increased financial burden. The delay in treatment escalates the severity of the problem, resulting in higher treatment costs. Patients might end up needing multiple hospital visits, which not only costs more but also affect their daily routines and work obligations.
iii **Loss of Teeth and Functionality:** Severe delays sometimes lead to irreparable damages, resulting in the loss of teeth that could have been saved with earlier interventions. Missing teeth affect chewing ability, restrict food options, and cause embarrassment or social discomfort due to changes in appearance, ultimately impacting confidence and self-esteem.
iv **Persistent Pain and Discomfort:** Delaying treatment often leads to persistent discomfort and pain. This ongoing discomfort affects sleep, eating habits, and overall quality of life.

The following excerpts were obtained from patient interviews:

*“Problems worsen over time”*

*“I pay more because I stayed home for long and problem is worse”*

*“It is embarrassing when it is mouth odour and I delay going to the hospital”*

*“My teeth problems make me less confident”*

*“There is persistent pain from toothache making it difficult to sleep”*

B **Impact Of Challenges On Patients Overall Experience At The Dental Clinic**

Experiences at the dental clinic reveal a comprehensive range of issues that significantly impacted the overall patient experience. The six themes are highlighted below:

i **Long Waiting Hours and Frustration**: Waiting for prolonged periods at the clinic was a major source of frustration. The excessive waiting times caused significant discomfort and dissatisfaction, leading to wasted time, missed work or school, and heightened anxiety as the appointment time drew nearer.
ii **Worsening Health Conditions and Increased Costs:** The delay in treatment aggravated the existing dental problem, causing it to worsen over time. This deterioration led to increased treatment costs and a sense of anxiety about seeking further dental treatments due to the perceived financial burden.
iii **Emotional and Psychological Distress:** The prolonged waiting times and discomfort during procedures induced emotional distress, leading to elevated blood pressure, heightened anxiety, and a pervasive sense of being on edge throughout the entire treatment period. The experiences led to feelings of fear, discomfort, and embarrassment, negatively impacting the patient’s mental well-being.
iv **Lack of Clarity in Communication and Treatment:** The reported misunderstandings about treatment plans and costs led to unmet expectations. This lack of clarity and unsatisfactory answers to questions, left the patient feeling uncertain, dissatisfied, and disappointed with the overall outcome.
v **Negative Interactions and Lack of Confidence:** Unpleasant encounters with staff members, including arguments and perceived disrespect, left patients feeling humiliated, embarrassed, and disrespected. Some patients felt exposed and uneasy during the procedure, impacting their confidence and comfort throughout the treatment.
vi **Avoidance of Recommended Procedures and Concerns about Hygiene:** Due to the discomfort experienced and lack of satisfaction with the provided treatment, patients chose to avoid recommended procedures, which in turn contributed to the worsening of their condition. Concerns about cross-infection and the number of individuals handling the patient for studies or training purposes caused additional distress and unease.

The following excerpts were obtained from patient interviews:

*“Waiting for long hours was frustrating”*

*“I became anxious for the appointment”*

*“My problem worsened with time”*

*“I felt the student doctors were more interested in using me for their requirements”*

*“I was scared for a cross-infection on the ward”*

C **Patients Coping Mechanisms Used To Address Challenges Faced During Dental Visits**

Patient responses were grouped into five themes. This composite study investigated the various coping mechanisms patients employed in addressing various challenges they mentioned earlier.

i **Appointment Management:** Some patients proactively managed their appointments by contacting the doctor directly to inquire about available times, and specifically requesting either early morning or late afternoon appointments. Additionally, visiting the clinic days in advance to book appointments showed an attempt to secure suitable timings.
ii **Communication and Anxiety Management**: Expressing concerns directly to the dental staff by disclosing anxiety and fear and employing techniques such as deep breathing to manage anxiety and fear demonstrated a proactive approach to addressing emotional distress during dental visits by respondents.
iii **Seeking Clarifications and Detailed Explanations**: Actively seeking clarity on the treatment plan by asking the dentist to explain in detail, and requesting further clarification if some aspects were unclear or confusing, represented the constructive approach respondents used towards understanding the treatment process.
iv **Assertiveness in Addressing Issues:** The patients showed assertiveness by expressing concerns directly to the staff and speaking back when they felt disrespected. Additionally, opting for other clinics or specialist services in specific instances were some paths some patients treaded.
v **Seeking Second Opinions and Reporting Concerns**: Some patients sought second or third opinions from other facilities and decided to try alternative clinics after facing dissatisfaction. Also, reporting concerns to the directorate management was an approach that was used as well.

The following excerpts were obtained from patient interviews:

*“I called the doctor to ask of available times”*

*“I told the dental staff I had anxiety and fear”*

*“I sent a report to the directorate management”*

*“I spoke back when disrespected”*

*“I opted for other clinics and came back here for specialist services”*

D **Patients’ Suggestions On Addressing Challenges And Improving Overall Patient Experience At The Dental Clinic**

This section of composite study looked into what patients thought could be done to address challenges mentioned. Their responses were grouped into six themes as highlighted below:

i **Improved Appointment Systems:** Offering same-day appointments and implementing online appointment systems can significantly reduce waiting times, providing patients with more flexibility and convenience in securing appointments.
ii **Training and Techniques for Anxiety Reduction**: Staff training in techniques that help in calming patients down is crucial. Creating a supportive environment that helps alleviate anxiety and stress during procedures is beneficial for patient comfort.
iii **Communication and Patient-Centred Care:** Encouraging a culture of community within the clinics, using simple language instead of medical jargon, and advocating for patient participation by encouraging questions and concerns will enhance communication and foster a patient-centred approach in dental care.
iv **Pain-Free Procedures and Privacy:** A key suggestion is to focus on making dental procedures less painful, which can significantly improve the patient experience. Ensuring fewer individuals are present in the treatment room during procedures, and providing partitioned wards can enhance privacy and comfort during treatment.
v **Community Outreach and Access to Care:** Extending dental services to the community through outreach programs, raising awareness about oral health, and offering treatments in community settings can improve access to care, particularly for underprivileged or remote areas.
vi **Financial Assistance and Treatment Options:** Prioritizing poor communities, offering treatments on credit, reducing costs for certain treatment options, and providing comfortable payment plans can address financial barriers, making dental care more accessible and affordable for a wider range of patients.

The following excerpts were obtained from patient interviews:

“*Clinics should offer same day appointments”*

*“There should be online appointment systems”*

*“Staff should stop the use of the big terms.”*

*“The wards should be partitioned”*

*“There should be reduced cost for some treatment options”*

*“Patients should have a comfortable payment plan”*

E **Patients’ Suggestions On How Dental Professionals Can Improve Communication And Transparency With Patients To Address Patient Concerns Effectively.**

The patients’ suggestions aimed at improving communication and transparency between dental professionals or healthcare providers and patients are detailed in the four themes below.

i **Use of Layman’s Terms and Visual Aids:** Utilizing jargon-free language, avoiding complex dental terminologies, and explaining procedures in plain language can enhance patient understanding. Visual aids, such as diagrams, videos, and models, can significantly aid in explaining conditions and procedures in a more accessible manner, making the information clearer and easier to comprehend for patients.
ii **Documented Treatment Plans and Information**: Providing a document detailing treatment plans and procedures to patients ensures that, they have written information for reference and understanding. Offering brochures detailing clinic facilities and treatments during the waiting period further informs and educates patients about available services.
iii **Utilizing Technological Communication**: Sending appointment reminders through text messages or emails serves as a practical approach to keep patients informed and engaged. It helps in reducing missed appointments and ensures patients are adequately prepared for their scheduled visits.
iv **Improved Staff Communication**: Staff displaying attentiveness by not using personal devices while communicating with patients and actively engaging and showing signs of concern when listening can enhance patient-staff interactions and improve overall communication experiences.

The following excerpts were obtained from patient interviews:

*“Use plain languages to explain procedure”*

*“Brochures on clinic and treatments offered should be provided while waiting”*

*“Send patients e-mails prior to appointments”*

*“Staff should not be on their phones and tablets while communicating with patients”*

*“When listening, staff should show signs they are concerned”*

F **Effects Of Addressing Challenges On Overall Oral Health And Well-being of Patients**

The patients’ responses highlight several ways in which addressing challenges can positively impact the oral health and overall well-being of dental patients which is captured in the four themes below.

i **Improved Treatment Understanding and Compliance**: If patients understand the procedures and treatment plans, they are more likely to comply with the recommended treatments, ensuring they follow through with necessary care, and thus effectively managing their oral health conditions.
ii **Enhanced Overall Health and Disease Prevention**: Better oral health practices through regular visits can lead to improved overall health and prevent the progression of oral diseases. Regular check-ups can help in early detection and management of potential issues, thereby reducing the risk of diseases worsening, and improving the patient’s overall health.
iii **Increased Patient Satisfaction and Confidence**: Addressing challenges would lead to higher patient satisfaction, encouraging patients to attend follow-up visits and promoting confidence in seeking dental care. This will ensure patients are more likely to engage in regular dental visits, thereby maintaining their oral health and reducing the possibility of complications.
iv **Financial Impact**: If oral diseases are prevented from worsening due to regular visits and proper management, it can significantly reduce treatment costs for patients, thereby lessening the financial burden associated with extensive procedures and advanced diseases.

The following excerpts were obtained from patient interviews

*“I will be compliant to treatment if I understand what is done for me”*

*“In the long run my overall health will be improved”*

*“If I am satisfied, I will turn up for all other procedures and recommend the clinic to my friends and family”*

*“Diseases will not worsen so less money will be spent on treatment”*

*“If I do not have any concerns to care, dentists will not do extensive procedures on me”*

*“Seeking regular visits will prevent the progression of diseases”*

**G. Patients’ Suggestions On Overcoming Barriers To Make It Easier To Access Dental Care**

In this section, patients talked about various ways they could overcome barriers. Their responses were summarized in five themes as seen in this composite study.

a **Funding:**
i Increased government subsidies and funding for dental care can make it more affordable and accessible, especially for underserved populations.
ii Dental Savings Plans: Offering dental savings plans that allow individuals to save specifically for dental care could reduce financial barriers.
iii Lower Cost at Student Clinics: Allowing student clinics to provide dental care services at a reduced cost could make it more affordable for individuals, while also providing training opportunities for students.
b **Encouraging Preventive Care:**
i Patient Education and Communication: Enhancing patient education about oral health and the importance of regular dental visits can help reduce fear and anxiety.
ii Mobile Dental Clinics and Outreaches: Offering dental care through mobile clinics can reach underserved areas and populations with limited access.
iii Telehealth and Teledentistry: Implementing remote dental consultations and treatments can expand access to care, especially in remote or rural areas.
c **Rewards:**
i Positive Reinforcement: Recognizing and rewarding individuals who regularly attend dental appointments could encourage continued engagement with dental care.
ii Desensitization and Gradual Exposure: Implementing strategies that gradually expose individuals to dental environments and procedures can help reduce anxiety and fear.
d **Pain Management Strategies:**
i Ensuring effective pain management during dental procedures can alleviate fear and discomfort.
ii More Pediatric-Friendly Approaches: Creating child-friendly and welcoming dental environments can help alleviate fear among young patients
e **Appointments and time:**
i Dental Home Visits: Offering dental care services at home for individuals who have difficulty traveling to a clinic.
ii Extended and Reduced Clinic Hours: Adapting clinic hours to accommodate different schedules can make it easier for individuals to seek dental care.
iii Online Appointment Booking: Providing the convenience of booking appointments online can simplify the process.
iv Same-Day Appointments and Emergency Dental Services: Offering same-day appointments and emergency services can address urgent dental needs promptly.
v Patient Reminder Systems: Implementing systems to remind patients of their upcoming appointments can reduce no-show rates and encourage attendance.

The following excerpts were obtained from patient interviews Table 3:

**Table 3:** Relationship between patient demographics, perceived treatment seeking behaviours and quality of dental services.

Hypothesis	x²	df	p = 0.05	Cramer’s
**H1:Demographics and Perceived** **Treatment Seeking Behaviours**				
Age	25.681	18	0.107	0.107
Gender	1.382	3	0.710	0.710
**Marital Status**	21.197	9	**0.012**	**0.012**
**Education**	30.976	15	**0.009**	**0.009**
Religion	11.288	9	0.256	0.256
Occupation	19.445	18	0.365	0.365
**H2:Demographics and Perceived** **Quality of Dental Services**				
Age	18.636	12	0.098	0.098
**Gender**	15.420	2	**0.000**	**0.000**
**Marital Status**	12.322	6	**0.055**	**0.055**
Education	11.790	10	0.299	0.299
Religion	2.006	4	0.735	0.735
Occupation	14.701	12	0.258	0.258

*Source: Survey, 2023.*

*“Government should reduce cost of dental care”*

*“The dental clinic should provide an avenue of gradual savings to make us more interested in dental care and to ease us when we come to the dental clinic”*

*“The prices at the student clinics should be less than the ones at the main clinic. This is a win-win situation for me the patient and the students using me for their studies”*

*“There should be more outreaches”*

*“Dental care should come at our homes”*

*“Desensitization and Gradual Exposure”*

*“Pain is too much to handle during treatment”*

*“Sometimes staff are stern and unfriendly to my kids”*

*“Sometimes I come to meet the clinic closing so, if possible, they should extend the time”*

## Discussions

The study explores the multifaceted challenges dental patients face, both before and during their visits to the Oral Health Directorate of Komfo Anokye Teaching Hospital (KATH). By analyzing these barriers and their impacts, the study uncovers crucial insights into the factors influencing dental care access and utilization in Ghana.

### Demographic influences on dental care behaviour

The findings reveal that most respondents were female and between the ages of 25–34, with marital status and educational level statistically influencing treatment-seeking behaviours. Gender also emerged as a significant determinant of the quality of care sought. These demographic variables underscore disparities in dental care access, indicating the need for tailored interventions that consider socio-demographic contexts [10–13]. Higher levels of education were associated with better treatment-seeking behaviour (p = 0.009), suggesting that knowledge and awareness play crucial roles in health-seeking decisions. Gender disparities pointed to systemic barriers that limit access, while marital status influenced both the frequency and quality of care sought (p = 0.012). For instance, married individuals might prioritize family obligations over personal health, while single respondents may have fewer financial and social constraints.

### Challenges faced before seeking dental care

More than half of the respondents reported encountering barriers prior to seeking dental care. Key challenges included high costs, limited availability of appointment times, and inadequate insurance coverage [12,14]. Fear and anxiety, mobility issues, and negative past experiences further compounded the problem [11,15]. These barriers had cascading effects. Patients who delayed seeking care often experienced worsening oral health, leading to higher treatment costs and persistent discomfort. Financial burdens escalated as untreated conditions became more severe, requiring complex and expensive interventions. Psychological effects, such as stress and reduced confidence, also emerged, reflecting the far-reaching consequences of delayed care [16,17]. These findings mirror global trends where financial and systemic barriers disproportionately affect low-income populations [14,15].

### Barriers during dental clinic visits

Challenges persisted even during dental visits, with two-thirds of respondents reporting difficulties. Dental anxiety was the most prevalent concern, affecting 77% of respondents, followed by lengthy waiting times, lack of privacy, and negative interactions with dental staff [13,14]. Many patients described waiting times as a significant source of frustration, leading to wasted time, missed work, and heightened anxiety. Inadequate communication and transparency during visits compounded these frustrations, leaving patients uncertain about their treatment plans and costs. The teaching hospital setting added another layer of complexity, as multiple observers during procedures made patients feel exposed and uncomfortable [18]. Such experiences highlight the need for better patient-staff interactions and streamlined clinical processes.

### Relationship between background characteristics and treatment-seeking behaviour

The relationship between demographic characteristics and treatment-seeking behaviour revealed critical insights. Educational level was positively associated with better health-seeking behaviour (p = 0.009), suggesting that higher education enhances awareness and prioritization of oral health [15,16]. Marital status also influenced treatment patterns; married respondents were more likely to postpone care, possibly due to competing priorities or financial constraints (p = 0.012). Gender disparities were evident, with men less likely to seek preventive care compared to women, aligning with previous findings that males often delay healthcare until conditions become severe [13]. These dynamics underscore the importance of addressing socio-demographic factors to improve access and utilization.

### Relationship between demographics and quality of care sought

Gender and marital status were significantly associated with the quality of care sought (p = 0.000 and p = 0.055, respectively). Women were more likely to engage with and report higher satisfaction in care processes, while men exhibited more reactive patterns of care-seeking. Similarly, married individuals experienced varied care quality, potentially reflecting household responsibilities or differing expectations of care [13,14]. This correlation highlights the nuanced interplay of personal and systemic factors in determining care outcomes.

### Communication and transparency

While the majority of respondents were somewhat satisfied with communication from dental providers, nearly a third expressed dissatisfaction. Patients reported that unclear explanations, the use of medical jargon, and insufficient engagement exacerbated their challenges [18]. Improved communication is essential for building trust, reducing anxiety, and ensuring adherence to treatment plans. Respondents emphasized the importance of empathetic interactions and the use of plain language to explain procedures and options. Providing visual aids and detailed treatment plans could further enhance understanding and satisfaction, bridging gaps in patient-provider communication [10,19,20].

### Impact of barriers on oral health and well-being

Delays in seeking care due to barriers had profound consequences. Patients reported deterioration of oral health, leading to more severe conditions that required extensive and costly treatments. Emotional and psychological distress, including anxiety and reduced self-esteem, were common outcomes. Loss of teeth and diminished functionality further compounded these issues, affecting patients’ ability to eat, speak, and interact socially [16,21]. The cyclical nature of these impacts was evident; delays caused by barriers often led to worsened conditions, further reinforcing the financial and emotional burdens of care [17,20].

### Relationship between quantitative and qualitative findings

The study’s quantitative and qualitative findings provided complementary insights. Quantitatively, 55.4% of respondents identified barriers to care, with cost (100%), appointment availability (100%), and insurance (72.2%) as the top challenges. These figures were expanded upon qualitatively, where respondents described the cascading effects of these barriers, such as worsened oral health, increased costs, and emotional strain. Dental anxiety, reported by 77% of respondents in quantitative findings, was explored qualitatively as both a driver and result of negative experiences during care, creating a feedback loop that discouraged future visits [14,15]. Communication gaps, identified by 29.2% of respondents, were further elaborated qualitatively, revealing how unclear explanations and perceived indifference compounded dissatisfaction [19,20].

### Recommendations for improvement

Respondents suggested several measures to improve dental care experiences, such as implementing same-day and online appointment systems, training staff in anxiety reduction techniques, and enhancing privacy during treatments. Expanding community outreach and offering financial assistance were also identified as critical steps to improve access, particularly for underserved populations [18,21]. Additionally, respondents emphasized the importance of preventive care and patient education. Providing resources to demystify dental procedures and highlighting the benefits of regular check-ups could help mitigate anxiety and encourage proactive care-seeking behaviour.

### Strengths and limitations

Participant selection where a fifth of OPD patients were selected to undertake this study gave an advantage of diversity, good representation and distribution. The study did not consider the views of healthcare practitioners with respect to perceived barriers. It cannot therefore be assumed that these barriers are a fair representation of all barriers in the dental setting.

### Conclusions

The study highlights significant barriers and challenges patients face in accessing dental care. More than half of respondents had encountered barriers before seeking dental care, primarily cost and the lack of available appointment times, which were ranked as the most common challenges. The repercussions of these barriers were quite significant with most respondents reporting that the barriers led to the delay or avoidance of seeking dental care. The study further highlighted various challenges encountered by patients while waiting for dental care, with two-thirds of respondents reporting difficulties during their visits. These challenges included dental anxiety or fear, lengthy waiting times, lack of privacy during treatment, negative interactions with dental staff, high treatment costs, and hygiene concerns. The results of this study revealed a statistically relevant association between demographics and perceived treatment-seeking behaviours with gender, marital status and education exhibiting a strong correlation with the perceived treatment seeking behaviours and quality of care sought. This gender-based difference could have important implications for understanding and addressing disparities in dental care access and utilization. The findings underscore the importance of a holistic approach to tackling barriers, ensuring equitable access to dental care for all [14,19,21].

### Recommendations

This study makes it evident that when the numerous challenges patients face are worked on, overall oral health outcome improves and patient satisfaction is achieved. These are recommendations for healthcare providers, policymakers, and dental care professionals. These recommendations were suggested by patients during this study. However, they can be reiterated below: subsidies, tele dentistry, in lieu of waiting for cases to exacerbate before coming to the clinic, comprehensive patient education and preventive care should be well emphasized to all patients, improved appointment systems, minimal pain and better communication system. This study highlights the significant challenges faced by patients in accessing dental care in Ghana. These barriers include high costs, limited appointment availability, inadequate insurance coverage, anxiety, accessibility challenges, negative past experiences, and mobility issues. To improve oral health outcomes and ensure equitable access to dental care, the study recommends: implementing subsidies or financial assistance programs, tele-dentistry, prioritizing preventive care and patient education, improved appointment systems, implementing strategies to minimize pain and fostering open and transparent communication. By addressing these recommendations, Ghana can make significant strides in improving oral health outcomes and ensuring equitable access to dental care for all citizens.

## References

[1] Northridge ME, Kumar A, Kaur R. Disparities in Access to Oral Health Care. Annu Rev Public Health. 2020;41:513–35. doi: 10.1146/annurev-publhealth-040119-094318 31900100 PMC7125002

[2] Righolt AJ, Jevdjevic M, Marcenes W, Listl S. Global-, Regional-, and Country-Level Economic Impacts of Dental Diseases in 2015. J Dent Res. 2018;97(5):501–7. doi: 10.1177/0022034517750572 29342371

[3] Duangthip D, Chu CH. Challenges in Oral Hygiene and Oral Health Policy. Front Oral Health. 2020;1:575428. doi: 10.3389/froh.2020.575428 35047981 PMC8757757

[4] Marcenes W, Kassebaum NJ, Bernabé E, Flaxman A, Naghavi M, Lopez A, et al. Global burden of oral conditions in 1990-2010: a systematic analysis. J Dent Res. 2013;92(7):592–7. doi: 10.1177/0022034513490168 23720570 PMC4484374

[5] Kassebaum NJ, Bernabé E, Dahiya M, Bhandari B, Murray CJL, Marcenes W. Global burden of severe periodontitis in 1990-2010: a systematic review and meta-regression. J Dent Res. 2014;93(11):1045–53. doi: 10.1177/0022034514552491 25261053 PMC4293771

[6] Krishnan L, C. S. A, Kumar PDM. Barriers to access dental care services among adult population: A systematic review. JGOH. 2020;3:54–62. doi: 10.25259/jgoh_1_2020

[7] Saleh N, Elashri N, Mohamed H, El-Gilany A-H. Barriers Affecting the Utilization of Dental Health Services among Community Dwelling Older Adults. Alexandria Scientific Nursing Journal. 2018;20(1):103–18. doi: 10.21608/asalexu.2018.207750

[8] Bahadori M, Ravangard R, Asghari B. Perceived Barriers Affecting Access to Preventive Dental Services: Application of DEMATEL Method. Iran Red Crescent Med J. 2013;15(8):655–62. doi: 10.5812/ircmj.11810 24578831 PMC3918188

[9] Asthana G, Arora SA, Kalsi R, Saurav K. Barriers to seeking dental treatments. Journal Title Abbreviation. 2021;12(3):87–91.

[10] Borreani E, Wright D, Scambler S, Gallagher JE. Minimising barriers to dental care in older people. BMC Oral Health. 2008;8:7. doi: 10.1186/1472-6831-8-7 18366785 PMC2335092

[11] Ajayi D, Arigbede A. Barriers to oral health care utilization in Ibadan, South West Nigeria. Afr Health Sci. 2013;2013(4):507–13. 10.4314/ahs.v12i4.17 PMC3598293 23515140

[12] Malhi R, Basavaraj P, Singla A, Jankiram C, Pandita V, Vashishtha V. Perceived barriers in accessing dental care among patients attending dental institute using decision-making trial and evaluation laboratory method. J Indian Assoc Public Health Dent. 2015;13(2):152. doi: 10.4103/2319-5932.159052

[13] Damilare KA, Abass D, Antwi-Agyei D, Osei-Owusu F, Ahenkan E, Boadu KAO, Boadu RO. Patients Perceived Knowledge, Attitude, and Practice of Dental Abscess Management in Periurban District, Ghana. 2022. 10.1155/2022/2266347 PMC9236784 35769666

[14] Saleh N, Elashri N, Mohamed H, El-Gilany A-H. Barriers Affecting the Utilization of Dental Health Services among Community Dwelling Older Adults. Alexandria Scientific Nursing Journal. 2018;20(1):103–18. doi: 10.21608/asalexu.2018.207750

[15] Al-jabr A, Alhujaili A. Cost as a Barrier to Access Dental Healthcare in Saudi Patients. Oral Health and Dentistry. 2018;2(4):401-–409.

[16] Chideka K, Klass C, Dunne S, Gallagher JE. Listening to older adults: community consultation on a new dental service. Community Dent Health. 2015;32(4):231–6. 26738221

[17] Nagarjuna P, Reddy VcS, Sudhir K, Kumar RVSK, Gomasani S. Utilization of dental health-care services and its barriers among the patients visiting community health centers in Nellore District, Andhra Pradesh: A cross-sectional, questionnaire study. J Indian Assoc Public Health Dent. 2016;14(4):451. doi: 10.4103/2319-5932.195844

[18] Jain V, Sequeira P, Jain J, Chancy U, Maliyil M, SC B. Barriers in utilization of oral health care services among patients attending primary and community health centres in Virajpet, South Karnataka. Natl J Med Dent Res. 2013;1:33.

[19] Brennan MG. Confidentiality in practice; knowing when to keep a secret. Vital. 2010;7(3):44–6. doi: 10.1038/vital1192

[20] Henríquez-Tejo RB, Cartes-Velásquez RA. Patients’ perceptions about dentists: a literature review. Odontoestomatol. 2016;18(27):15–22.

[21] Ashraf S. Self reported oral Health and Oral Health Practices among Bangladeshi Immigrants In, Norway University of Oslo, The Faculty of Medicine Institute of Health and Society Department of Community Medicine 2017.

